# Poly(ester-anhydrides) Derived from Esters of Hydroxy Acid and Cyclic Anhydrides

**DOI:** 10.1021/acs.biomac.2c00542

**Published:** 2022-07-26

**Authors:** Yuvaraj Arun, Radhakanta Ghosh, Abraham J. Domb

**Affiliations:** The Alex Grass Center for Drug Design & Synthesis and the Center for Cannabis Research, School of Pharmacy, Institute of Drug Research, Faculty of Medicine, The Hebrew University of Jerusalem, Jerusalem 9112001, Israel

## Abstract

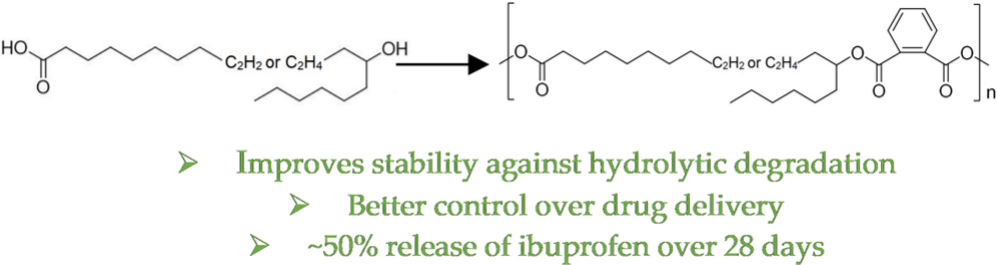

The alternating architecture and hydrophobic side chains hinder hydrolytic cleavage and anhydride interchange in poly(sebacic acid-ricinoleic acid) (P(SA-RA)), which provides stable polyanhydrides at room temperature. In this report, a series of polyanhydrides were designed to investigate the effect of ester bonds, hydrophobic side chains, phenyl moieties, and their distance from anhydride bonds on their stability and properties. Polyanhydrides with alternating architecture are constructed by the polymerization of ester-diacids prepared from ricinoleic or other hydroxy acids with anhydrides such as succinic, maleic, and phthalic anhydrides. The hydrophobic side chains are designed closer to anhydride bonds to investigate hindrance to hydrolytic cleavage and anhydride interchange. Polyanhydrides were obtained by the activation of ester-diacid using acetic anhydride followed by melt condensation. The reactions were monitored by NMR, Fourier transform infrared (FTIR), and gel permeation chromatography (GPC). The synthesized poly(ester-anhydride)s with a shorter chain length compared to P(SA-RA) were stable at room temperature. The hydrolytic degradation studies reveal that the phenyl moiety present in poly(ricinoleic acid phthalate) (PRAP) and poly(hydroxystearic acid phthalate) (PHSAP) reduces the hydrolysis of anhydride bonds. Poly(hydroxyoctanoic acid succinate) (PHOAS) demonstrates the highest molecular weight of all tested polymers. The results reveal that the presence of hydrophobic side chains, phenyl moieties, and their distance from anhydride bonds significantly improves the stability. These stable polyanhydrides can provide convenience to use in control drug-delivery applications. The *in vitro* drug release study using ibuprofen shows that polymers with aromatic units such as PRAP and PHSAP establish sustained release, which presents more than 50 and 40% of ibuprofen over a period of 28 days.

## 1 Introduction

Polyanhydrides have been investigated as carriers for the controlled delivery of several drugs.^1,2^ Polyanhydrides are desirable as controlled release carriers because of their surface eroding properties.^3^ Polyanhydrides have inherent high reactivity toward water, which prompts rapid hydrolytic degradation. Due to the high rate of hydrolysis, polyanhydrides endure surface erosion rather than bulk degradation. Gliadel wafer, an approved polyanhydride copolymer of carboxyphenoxy propane and sebacic acid, is a bioresorbable medicinal implant used to deliver carmustine, an anticancer agent to cerebral tumor sites.^4^ Polyanhydride-based particles have been widely studied in many formulations for effective drug delivery.^5−7^ Nevertheless, the number of polyanhydride products existing in the market is fewer compared to polyesters. Even though polyanhydrides are easy and inexpensive to synthesize and scale up, they exhibit a short shelf-life.^8,9^ Polyanhydrides are prone to hydrolytic degradation and depolymerization via anhydride interchange during storage.^10,11^ Hence, polyanhydrides need to be kept under freezing storage conditions that restrict their usage in drug-delivery products.

Alternating polyanhydride copolymers have been tested for their extended shelf-life. Poly(ester-anhydride)s exhibit a better drug release profile; however, the shelf-life of the polymer does not substantially improve.^12,13^ Polyanhydrides based on ε-caprolactone were found with enhanced hydrolytic stability with a limited shelf-life.^14,15^ Poly(ester-anhydride)s based on ricinoleic acid and sebacic acid have been studied and extensively employed in drug-delivery applications.^16−18^ We synthesized polyanhydrides with improved stability and shelf-life.^19^ This polyanhydride is synthesized from ricinoleic and sebacic acid with alternating ester-anhydrides and is stable at 25 °C for more than 18 months. The alternating architecture provides improved stabilization through the hydrophobic side chains by hindering hydrolytic cleavage and anhydride interchange.

The safety of polyester anhydrides, particularly poly(sebacic acid-ricinoleic acid) 30:70, is reported.^20^ The polymers degrade into their starting materials that are eliminated from the site of injection and the body.^21,22^

On the other hand, there is a growing demand for injectable systems using viscous low melting polymers that facilitate new potential uses for the polyanhydrides.^23^ The injectable formulation can be simply extruded using a needle that produces a depot under the aqueous atmosphere and gradually releases the loaded drug. This allows localized drug delivery with marginal invasion and extremely predictable rates of drug release.

In this report, we designed a series of new polyanhydrides with altered distance between the anhydride moiety and hydrophobic side chain to achieve better stability and control over drug release with injectable properties comparable to the previously reported P(SA-RA) polymer. We developed a novel methodology to construct polyanhydrides with tuneable properties by the reaction of ricinoleic or other hydroxy acids with anhydrides such as succinic, maleic, and phthalic anhydrides.

## 2 Experimental Section

### 2.1 Materials

Hydroxy acids such as 12-hydroxydodecanoic acid (HDDA, 97%, Aldrich), 2-hydroxyoctanoic acid (HOA, 98%, Alfa Aesar), and 12-hydroxystearic acid (HSA, 75%, TCI) were used as received. Diacids such as sebacic acid (SA, 99%) and dodecanedioic acid (DDDA, 99%) were purchased from Sigma-Aldrich and used as received. Anhydrides such as succinic anhydride (99%; Aldrich), maleic anhydride (99%; Sigma-Aldrich), phthalic anhydride (99%; Aldrich), and acetic anhydride (Merck) were purchased and used as received. Castor oil was purchased from Tamar (Jerusalem, Israel). All solvents and reagents (analytical-grade) were purchased (Sigma-Aldrich or BioLab) and used without further purification.

### 2.2 Spectral Analysis

Fourier transform infrared (FTIR) spectroscopy was performed using a Smart iTR ATR sampling accessory for a Nicolet iS10 spectrometer with a diamond crystal (Thermo Scientific, Massachusetts). ^1^H and ^13^C NMR spectra were obtained on Varian 300 and 75 MHz NMR spectrometers, respectively, in tubes with 5 mm outside diameters. CDCl_3_ or DMSO-*d*_6_ containing tetramethylsilane served as a solvent and shift reference. Thin layer chromatography (TLC) plates were purchased from Merck (Silica gel matrix coated with a fluorescent indicator on aluminum plates).

### 2.3 Molecular-Weight Determination

Molecular weights were determined by a gel permeation chromatography (GPC) system, Waters 1515. Isocratic high performance liquid chromatography (HPLC) pump with a Waters 2410 refractive index detector, a Waters 717 plus autosampler, and a Rheodyne (Cotati, CA) injection valve with a 20 μL loop. The samples were eluted with CHCl_3_ (HPLC grade) through a linear Styragel HR4E column (Waters) with a molecular-weight range of 50–100K Da at a flowrate of 1 mL/min. The molecular weights were determined relative to a polystyrene standards (Polyscience, Warrington, PA) calibration curve having weight-average molecular weight (*M*_w_) from 600 to 50 000.

### 2.4 Synthesis of Monomers

#### 2.4.1 Ricinoleic Acid (RA)

RA was prepared from the hydrolysis of castor oil as described above.^24^ Castor oil (200 g) was hydrolyzed by refluxing in a KOH (48 g) solution (ethanol, 400 mL) for 2 h. Double distilled water (DDW) (400 mL) was added to the reaction flask after the evaporation of ethanol. The clear yellowish solution was acidified with H_3_PO_4_ to reach pH ∼ 2. The fatty acid obtained was extracted with diisopropyl ether. The organic layer was dried over anhydrous Na_2_SO_4_, filtered, and evaporated to dryness. RA was obtained as a pale-yellow colored clear viscous liquid. 12-Hydroxyoctadec-9-enoic acid; ^1^H NMR (300 MHz, chloroform-*d*) δ 5.56 (dt, *J* = 10.8, 7.4 Hz, 1H), 5.47–5.30 (m, 1H), 3.62 (p, *J* = 6.1 Hz, 1H), 2.34 (t, *J* = 7.4 Hz, 2H), 2.21 (t, *J* = 6.9 Hz, 2H), 2.04 (q, *J* = 6.8 Hz, 2H), 1.63 (p, *J* = 7.2 Hz, 2H), 1.48–1.43 (m, 2H), 1.37–1.20 (m, 16H), 0.88 (t, *J* = 6.0 Hz, 3H); FTIR (cm^–1^) 3008, 2924, 2854, 1708, 1457, 1410, 1244.

#### 2.4.2 Ricinoleic Acid Succinate (RAS)

RAS was prepared by a previously reported method with modifications.^25^ A solution of RA (20.0 g, 67 mmol, 1.0 equiv) and succinic anhydride (8.1 g, 80 mmol, 1.2 equiv) in toluene (80 mL) was stirred at 90 °C. The reaction was monitored by TLC using hexane/ethyl acetate/acetic acid (80/30/1 v/v/v) as an eluent and vanillin stain to identify the spots. After the full conversion of RA, the reaction mixture was cooled to RT, and toluene was removed using a roto-evaporator. Then, water was added to the residue and stirred for 15 min. Subsequently, ethyl acetate was used for extraction, and the organic layer was washed three times with distilled water. Then, the organic layer was dried over anhydrous Na_2_SO_4_ and evaporated to dryness. RAS was obtained with 96% yield (25.7 g) as a pale-yellow colored viscous liquid that solidified to white solid at RT. 12-((3-Carboxypropanoyl)oxy)octadec-9-enoic acid; ^1^H NMR (300 MHz, CDCl_3_) δ 5.55–5.39 (m, 1H), 5.39–5.23 (m, 1H), 4.90 (p, *J* = 6.3 Hz, 1H), 2.67 (t, *J* = 6.1 Hz, 2H), 2.61 (t, *J* = 5.8 Hz, 2H), 2.35 (t, *J* = 7.4 Hz, 2H), 2.31–2.24 (m, 2H), 2.01 (q, *J* = 7.2 Hz, 2H), 1.71–1.59 (m, 2H), 1.59–1.46 (m, 2H), 1.35–1.23 (m, 16H), 0.87 (t, *J* = 6.0 Hz, 3H); FTIR (cm^–1^) 3008, 2925, 2855, 1732, 1707, 1458, 1411, 1169.

#### 2.4.3 Ricinoleic Acid Maleate (RAM)

A solution of RA (20.0 g, 67 mmol, 1.0 equiv) and maleic anhydride (7.9 g, 80 mmol, 1.2 equiv) in toluene (80 mL) was stirred at 90 °C. The reaction was monitored by TLC using hexane/ethyl acetate/acetic acid (80/30/1 v/v/v) as an eluent, and vanillin stain was used to identify the spots. After the full conversion of RA, the reaction mixture was cooled to RT, and toluene was removed using a roto-evaporator. Then, water was added to the residue and stirred for 15 min at 50 °C. Subsequently, ethyl acetate was used for extraction, and the organic layer was washed three times with distilled water. Then, the organic layer was dried over anhydrous Na_2_SO_4_ and evaporated to dryness. RAM was obtained with 94% yield (25.0 g) as a pale-orange colored viscous liquid. 12-((3-Carboxyacryloyl)oxy)octadec-9-enoic acid; ^1^H NMR (300 MHz, CDCl_3_) δ 11.41 (s, 2H), 6.39 (d, *J* = 12.5 Hz, 1H), 6.34 (d, *J* = 12.5 Hz, 1H), 5.59–5.41 (m, 1H), 5.40–5.24 (m, 1H), 5.02 (p, *J* = 6.3 Hz, 1H), 2.36 (t, *J* = 7.4 Hz, 4H), 2.02 (q, *J* = 7.3 Hz, 2H), 1.62 (q, *J* = 7.0 Hz, 4H), 1.33–1.25 (m, 16H), 0.88 (t, *J* = 6.0 Hz, 3H); FTIR (cm^–1^) 3011, 2925, 2855, 1705, 1645, 1411, 1247, 1214, 1168.

#### 2.4.4 Ricinoleic Acid Phthalate (RAP)

A solution of RA (20.0 g, 67 mmol, 1.0 equiv) and phthalic anhydride (11.9 g, 80 mmol, 1.2 equiv) in toluene (80 mL) was stirred at 90 °C. The reaction was monitored by TLC using hexane/ethyl acetate/acetic acid (80/30/1 v/v/v) as an eluent, and vanillin stain was used to identify the spots. After the full conversion of RA, the reaction mixture was cooled to RT, and toluene was removed using a roto-evaporator. Then, water was added to the residue and stirred for 15 min at 50 °C. Subsequently, ethyl acetate was used for extraction, and the organic layer was washed three times with distilled water. Then, the organic layer was dried over anhydrous Na_2_SO_4_ and evaporated to dryness. RAP was obtained with 88% yield (26.3 g) as a pale-orange colored viscous liquid. 2-(((17-Carboxyheptadec-9-en-7-yl)oxy)carbonyl)benzoic acid; ^1^H NMR (300 MHz, CDCl_3_) δ 7.84 (dd, *J* = 6.6, 2.3 Hz, 1H), 7.72 (dd, *J* = 6.9, 2.0 Hz, 1H), 7.58 (dt, *J* = 7.6, 5.9 Hz, 2H), 5.61–5.44 (m, 1H), 5.42–5.30 (m, 1H), 5.11 (p, *J* = 6.3 Hz, 1H), 2.47–2.28 (m, 4H), 2.08–2.00 (m, 2H), 1.64 (p, *J* = 5.8, 4.8 Hz, 4H), 1.34–1.25 (m, 16H), 0.86 (t, *J* = 6.3 Hz, 3H); FTIR (cm^–1^) 3009, 2925, 2854, 2667, 1701, 1600, 1580, 1455, 1411, 1284, 1125, 1073.

#### 2.4.5 Hydroxystearic Acid Succinate (HSAS)

A solution of 12-hydroxystearic acid (HAS) (20.0 g, 67 mmol, 1.0 equiv) and succinic anhydride (8.0 g, 80 mmol, 1.2 equiv) in toluene (80 mL) was stirred at 90 °C. The reaction was monitored by TLC using hexane/ethyl acetate/acetic acid (80/30/1 v/v/v) as an eluent, and vanillin stain was used to identify the spots. After the full conversion of HSA, the reaction mixture was cooled to RT, and toluene was removed using a roto-evaporator. Water was then added to the residue and stirred for 15 min. Subsequently, ethyl acetate was used for extraction, and the organic layer was washed three times with distilled water. Then, the organic layer was dried over anhydrous Na_2_SO_4_ and evaporated to dryness. HSAS was obtained with 95% yield (25.4 g) as a white solid. 12-((3-Carboxypropanoyl)oxy)octadecanoic acid; ^1^H NMR (300 MHz, CDCl_3_) δ 4.90 (p, *J* = 6.3 Hz, 1H), 2.74–2.65 (m, 2H), 2.65–2.57 (m, 2H), 2.34 (t, *J* = 7.2 Hz, 2H), 1.63 (q, *J* = 7.1 Hz, 2H), 1.59–1.41 (m, 6H), 1.28–1.25 (m, 20H), 0.88 (t, *J* = 6.0 Hz, 3H); FTIR (cm^–1^) 2922, 2853, 1708, 1466, 1411, 1380, 1343, 1288, 1170.

#### 2.4.6 Hydroxystearic Acid Maleate (HSAM)

A solution of HSA (20.0 g, 67 mmol, 1.0 equiv) and maleic anhydride (7.8 g, 80 mmol, 1.2 equiv) in toluene (80 mL) was stirred at 90 °C. The reaction was monitored by TLC using hexane/ethyl acetate/acetic acid (80/30/1 v/v/v) as an eluent, and vanillin stain was used to identify the spots. After the full conversion of HSA, the reaction mixture was cooled to RT, and toluene was removed using a roto-evaporator. Then, water was added to the residue and stirred for 15 min at 50 °C. Subsequently, ethyl acetate was used for extraction, and the organic layer was washed three times with distilled water. Then, the organic layer was dried over anhydrous Na_2_SO_4_ and evaporated to dryness. HSAM was obtained with 92% yield (24.5 g) as a white solid. 12-((3-Carboxyacryloyl)oxy)octadecanoic acid; ^1^H NMR (300 MHz, CDCl_3_) δ 6.40 (d, *J* = 12.0 Hz, 1H), 6.35 (d, *J* = 12.0 Hz, 1H), 5.01 (p, *J* = 6.2 Hz, 1H), 2.34 (t, *J* = 7.3 Hz, 2H), 1.67–1.54 (m, 6H), 1.31–1.23 (m, 22H), 0.87 (t, *J* = 6.0 Hz, 3H); FTIR (cm^–1^) 3012, 2924, 2854, 1704, 1645, 1456, 1411, 1379, 1216, 1170.

#### 2.4.7 Hydroxystearic Acid Phthalate (HSAP)

A solution of HSA (20.0 g, 67 mmol, 1.0 equiv) and phthalic anhydride (11.8 g, 80 mmol, 1.2 equiv) in toluene (80 mL) was stirred at 90 °C. The reaction was monitored by TLC using hexane/ethyl acetate/acetic acid (80/30/1 v/v/v) as an eluent, and vanillin stain was used to identify the spots. After the full conversion of HSA, the reaction mixture was cooled to RT, and toluene was removed using a roto-evaporator. Then, water was added to the residue and stirred for 15 min at 50 °C. Subsequently, ethyl acetate was used for extraction, and the organic layer was washed three times with distilled water. Then, the organic layer was dried over anhydrous Na_2_SO_4_ and evaporated to dryness. HSAP was obtained with 90% yield (26.8 g) as a white solid. 2-(((17-Carboxyheptadecan-7-yl)oxy)carbonyl)benzoic acid; ^1^H NMR (300 MHz, CDCl_3_) δ 7.88 (d, *J* = 7.3 Hz, 1H), 7.66 (d, *J* = 7.4 Hz, 1H), 7.63–7.46 (m, 2H), 5.13 (p, *J* = 6.2 Hz, 1H), 2.36 (t, *J* = 7.3 Hz, 2H), 1.77–1.54 (m, 6H), 1.49–1.25 (m, 22H), 0.85 (t, *J* = 6.8 Hz, 3H); FTIR (cm^–1^) 3010, 2924, 2854, 1699, 1600, 1580, 1491, 1455, 1411, 1283, 1126, 1073.

#### 2.4.8 Hydroxyoctanoic Acid Succinate (HOAS)

A solution of 2-hydroxyoctanoic acid (HOA) (5.0 g, 31 mmol, 1.0 equiv) and succinic anhydride (3.8 g, 38 mmol, 1.2 equiv) in toluene (25 mL) was stirred at 90 °C. The reaction was monitored by TLC using hexane/ethyl acetate/acetic acid (80/30/1 v/v/v) as an eluent, and vanillin stain was used to identify the spots. After the full conversion of HOA, the reaction mixture was cooled to RT, and toluene was removed using a roto-evaporator. Then, water was added to the residue and stirred for 15 min. Subsequently, ethyl acetate was used for extraction, and the organic layer was washed three times with distilled water. Then, the organic layer was dried over anhydrous Na_2_SO_4_ and evaporated to dryness. HOAS was obtained with 92% yield (8.1 g) as a white solid. 2-((3-Carboxypropanoyl)oxy)octanoic acid; ^1^H NMR (300 MHz, CDCl_3_) δ 4.93 (t, *J* = 6.5 Hz, 1H), 2.93–2.77 (m, 2H), 2.65–2.45 (m, 2H), 1.87 (q, *J* = 6.6 Hz, 2H), 1.53–1.38 (m, 2H), 1.38–1.25 (m, 6H), 0.88 (t, *J* = 6.0 Hz, 3H); FTIR (cm^–1^) 3038, 2955, 2927, 2859, 1712, 1421, 1378, 1211, 1162.

#### 2.4.9 Hydroxydodecanoic Acid Succinate (HDDAS)

A solution of 12-hydroxydodecanoic acid (HDDA) (5.0 g, 23 mmol, 1.0 equiv) and succinic anhydride (2.8 g, 28 mmol, 1.2 equiv) in toluene (25 mL) was stirred at 90 °C. The reaction was monitored by TLC using hexane/ethyl acetate/acetic acid (80/30/1 v/v/v) as an eluent, and vanillin stain was used to identify the spots. After the full conversion of HDDA, the reaction mixture was cooled to RT, and toluene was removed using a roto-evaporator. Then, water was added to the residue and stirred for 15 min. Subsequently, ethyl acetate was used for extraction, and the organic layer was washed three times with distilled water. Then, the organic layer was dried over anhydrous Na_2_SO_4_ and evaporated to dryness. HDDAS was obtained with 91% yield (6.7 g) as a white solid. 12-((3-Carboxypropanoyl)oxy)dodecanoic acid; ^1^H NMR (300 MHz, CDCl_3_) δ 4.12 (t, *J* = 6.4 Hz, 2H), 2.75–2.66 (m, 2H), 2.66–2.57 (m, 2H), 2.36 (t, *J* = 6.9 Hz, 2H), 1.73–1.55 (m, 4H), 1.45–1.19 (m, 14H); FTIR (cm^–1^) 3038, 2916, 2850, 1724, 1690, 1419, 1312, 1238, 1212, 1176.

### 2.5 Synthesis of Polymers

#### 2.5.1 Poly(sebacic acid) (PSA)

PSA was synthesized by reflux of sebacic acid with acetic anhydride (1:5 w/v) followed by polymerization through melt condensation. PSA was synthesized by refluxing sebacic acid (50 g) with acetic anhydride (250 mL, 1:5 w/v) for 30 min with constant stirring.^26^ Excess acetic anhydride was evaporated to dryness under vacuum. The clear residue was further polymerized by melt condensation at 160 °C for 4 h under vacuum (10 mbar) with constant stirring. PSA was obtained as a pale-yellow solid. *M*_w_ by GPC = 10 600 (dispersity = 2.2); ^1^H NMR (300 MHz, chloroform-*d*) δ 2.45 (t, *J* = 7.4 Hz, 4H), 1.65 (p, *J* = 7.1 Hz, 4H), 1.44–1.22 (m, 8H); FTIR (cm^–1^) 2927, 2913, 2850, 1808, 1741, 1471, 1411, 1358, 1035.

#### 2.5.2 Poly(dodecanedioic acid) (PDDDA)

PDDA was synthesized by reflux of dodecanedioic acid with acetic anhydride (1:5 w/v) followed by polymerization through melt condensation. PDDDA was synthesized by refluxing dodecanedioic acid (50 g) with acetic anhydride (250 mL, 1:5 w/v) for 30 min with constant stirring. Excess acetic anhydride was evaporated to dryness under vacuum. The clear residue was further polymerized by melt condensation at 160 °C for 4 h under vacuum (10 mbar) with constant stirring. PDDDA was obtained as a pale-yellow solid. *M*_w_ by GPC = 12 000 (dispersity = 2.3); ^1^H NMR (300 MHz, CDCl_3_) δ 2.44 (t, *J* = 7.4 Hz, 4H), 1.65 (p, *J* = 7.2 Hz, 4H), 1.43–1.16 (m, 12H); FTIR (cm^–1^) 2915, 2849, 1804, 1740, 1472, 1410, 1333, 1265, 1068, 1030.

#### 2.5.3 Poly(sebacic acid-ricinoleic acid) (P(SA-RA))

P(SA-RA) was synthesized using PSA and RA by 30 and 70% weight ratios, respectively. PSA (15 g) and RA (35 g) were melted and stirred at 175 °C under inert nitrogen atmosphere.^19^ The molten mixture was kept for 24 h under inert atmosphere until no free RA remained in the reaction mixture. After 24 h, acetic anhydride (250 mL, 1:5 w/v) was added and refluxed at 140 °C for 30 min. Excess acetic anhydride was evaporated under vacuum at 70 °C. The residue was then subjected to melt condensation at 160 °C under vacuum (10 mbar) for 6 h. P(SA-RA) was obtained as a pale-yellow colored clear pasty polymer. *M*_w_ by GPC = 11 500 (dispersity = 2.3); ^1^H NMR (300 MHz, CDCl_3_) δ 5.54–5.40 (m, 1H), 5.40–5.26 (m, 1H), 4.88 (p, *J* = 6.3 Hz, 1H), 2.45 (t, *J* = 7.4 Hz, 2H), 2.32–2.21 (m, 4H), 2.02 (q, *J* = 7.7, 7.0 Hz, 2H), 1.77–1.44 (m, 8H), 1.37–1.21 (m, 26H), 0.87 (t, *J* = 6.0 Hz, 3H); FTIR (cm^–1^) 2925, 2854, 1817, 1731, 1464, 1412, 1376, 1174, 1031.

#### 2.5.4 Poly(sebacic acid-hydroxystearic acid) (P(SA-HSA))

P(SA-HSA) was synthesized using PSA and HSA by 30 and 70% weight ratios, respectively. PSA (15 g) and HSA (35 g) were melted and stirred at 175 °C under inert nitrogen atmosphere. The molten mixture was kept for 24 h under inert atmosphere until no free HSA remained in the reaction mixture. After 24 h, acetic anhydride (250 mL, 1:5 w/v) was added and refluxed at 140 °C for 30 min. Excess acetic anhydride was evaporated under vacuum at 70 °C. The residue was then subjected to melt condensation at 160 °C under vacuum (10 mbar) for 6 h. P(SA-HSA) was obtained as a pale-yellow colored clear pasty polymer. *M*_w_ by GPC = 13 100 (dispersity = 2.1); ^1^H NMR (300 MHz, CDCl_3_) δ 4.86 (p, *J* = 6.3 Hz, 1H), 2.44 (t, *J* = 7.4 Hz, 2H), 2.28 (t, *J* = 7.5 Hz, 2H), 1.78–1.53 (m, 6H), 1.53–1.41 (m, 4H), 1.41–1.12 (m, 32H), 0.87 (t, *J* = 5.8 Hz, 3H); FTIR (cm^–1^) 2923, 2852, 1815, 1730, 1465, 1412, 1377, 1175, 1032.

#### 2.5.5 Poly(ricinoleic acid succinate) (PRAS)

PRAS was synthesized by reflux of RAS with acetic anhydride followed by polymerization through melt condensation. PRAS was synthesized by refluxing RAS (10 g) with acetic anhydride (50 mL, 1:5 w/v) for 30 min with constant stirring. Excess acetic anhydride was evaporated to dryness under vacuum at 70 °C. The clear residue was further polymerized by melt condensation at 140 °C for 6 h under vacuum (10 mbar) with constant stirring. PRAS was obtained as a pale-yellow colored clear pasty polymer. *M*_w_ by GPC = 14 700 (dispersity = 1.83); ^1^H NMR (300 MHz, CDCl_3_) δ 5.54–5.40 (m, 1H), 5.39–5.23 (m, 1H), 4.89 (p, *J* = 6.3 Hz, 1H), 2.75 (t, *J* = 6.7 Hz, 2H), 2.63 (t, *J* = 7.1 Hz, 2H), 2.44 (q, *J* = 7.1 Hz, 2H), 2.35–2.21 (m, 2H), 2.01 (q, *J* = 6.5 Hz, 2H), 1.66 (p, *J* = 7.3 Hz, 2H), 1.59–1.45 (m, 2H), 1.32–1.23 (m, 16H), 0.87 (t, *J* = 6.0 Hz, 3H); FTIR (cm^–1^) 3010, 2925, 2855, 1819, 1732, 1463, 1410, 1182, 1037.

#### 2.5.6 Poly(ricinoleic acid maleate) (PRAM)

PRAM was synthesized by reflux of RAM with acetic anhydride followed by polymerization through melt condensation. PRAM was synthesized by refluxing RAM (10 g) with acetic anhydride (50 mL, 1:5 w/v) for 30 min with constant stirring. Excess acetic anhydride was evaporated to dryness under vacuum at 70 °C. The clear residue was further polymerized by melt condensation at 140 °C for 6 h under vacuum (10 mbar) with constant stirring. PRAS was obtained as a pale-brown colored clear pasty polymer. *M*_w_ by GPC = 11 900 (dispersity = 1.87); ^1^H NMR (300 MHz, CDCl_3_) δ 7.05–6.73 (m, 2H), 5.58–5.40 (m, 1H), 5.35–5.27 (m, 1H), 4.99 (p, *J* = 6.4 Hz, 1H), 2.54–2.41 (m, 2H), 2.36–2.26 (m, 2H), 2.01 (q, *J* = 7.7 Hz, 2H), 1.68–1.57 (m, 4H), 1.31–1.24 (m, 16H), 0.88 (t, *J* = 5.8 Hz, 3H); FTIR (cm^–1^) 3011, 2925, 2855, 1815, 1723, 1643, 1464, 1287, 1259, 1179, 1040.

#### 2.5.7 Poly(ricinoleic acid phthalate) (PRAP)

PRAP was synthesized by reflux of RAP with acetic anhydride followed by polymerization through melt condensation. PRAP was synthesized by refluxing RAP (10 g) with acetic anhydride (50 mL, 1:5 w/v) for 30 min with constant stirring. Excess acetic anhydride was evaporated to dryness under vacuum at 70 °C. The clear residue was further polymerized by melt condensation at 140 °C for 6 h under vacuum (10 mbar) with constant stirring. PRAS was obtained as a pale-brown colored clear pasty polymer. *M*_w_ by GPC = 8400 (dispersity = 1.86); ^1^H NMR (300 MHz, CDCl_3_) δ 7.84–7.74 (m, 1H), 7.74–7.65 (m, 1H), 7.63–7.48 (m, 2H), 5.46–5.40 (m, 1H), 5.34–5.28 (m, 1H), 4.87 (p, *J* = 6.4 Hz, 1H), 2.46–2.40 (m, 2H), 2.28–2.24 (m, 2H), 2.02–2.00 (m, 2H), 1.63–1.53 (m, 4H), 1.28–1.25 (m, 16H), 0.86 (t, *J* = 6.0 Hz, 3H); FTIR (cm^–1^) 3010, 2925, 2854, 1814, 1727, 1598, 1579, 1464, 1410, 1281, 1209, 1132, 1090, 1014.

#### 2.5.8 Poly(hydroxystearic acid succinate) (PHSAS)

PHSAS was synthesized by reflux of HSAS with acetic anhydride followed by polymerization through melt condensation. PHSAS was synthesized by refluxing HSAS (10 g) with acetic anhydride (50 mL, 1:5 w/v) for 30 min with constant stirring. Excess acetic anhydride was evaporated to dryness under vacuum at 70 °C. The clear residue was further polymerized by melt condensation at 140 °C for 6 h under vacuum (10 mbar) with constant stirring. PHSAS was obtained as a pale-yellow colored clear pasty polymer. *M*_w_ by GPC = 19 100 (dispersity = 2.44); ^1^H NMR (300 MHz, CDCl_3_) δ 4.88 (p, *J* = 6.3 Hz, 1H), 2.77 (t, *J* = 6.7 Hz, 2H), 2.65 (t, *J* = 6.4 Hz, 2H), 2.51–2.42 (m, 2H), 1.65 (p, *J* = 7.2 Hz, 2H), 1.52 (q, *J* = 6.5 Hz, 6H), 1.31–1.25 (m, 20H), 0.88 (t, *J* = 6.4 Hz, 3H); FTIR (cm^–1^) 2925, 2854, 1820, 1732, 1465, 1411, 1378, 1356, 1184, 1040.

#### 2.5.9 Poly(hydroxystearic acid maleate) (PHSAM)

PHSAM was synthesized by reflux of HSAM with acetic anhydride followed by polymerization through melt condensation. PHSAM was synthesized by refluxing HSAM (10 g) with acetic anhydride (50 mL, 1:5 w/v) for 30 min with constant stirring. Excess acetic anhydride was evaporated to dryness under vacuum at 70 °C. The clear residue was further polymerized by melt condensation at 140 °C for 6 h under vacuum (10 mbar) with constant stirring. PHSAM was obtained as a pale-brown colored clear pasty polymer. *M*_w_ by GPC = 23 600 (dispersity = 2.69); ^1^H NMR (300 MHz, CDCl_3_) δ 6.96–6.82 (m, 1H), 6.36–6.25 (m, 1H), 4.98 (p, *J* = 5.9 Hz, 1H), 2.57–2.38 (m, 2H), 1.71–1.52 (m, 6H), 1.43–1.24 (m, 22H), 0.87 (t, *J* = 6.0 Hz, 3H); FTIR (cm^–1^) 3012, 2924, 2854, 1815, 1720, 1640, 1464, 1394, 1284, 1223, 1181, 1037.

#### 2.5.10 Poly(hydroxystearic acid phthalate) (PHSAP)

PHSAP was synthesized by reflux of HSAP with acetic anhydride followed by polymerization through melt condensation. PHSAP was synthesized by refluxing HSAP (10 g) with acetic anhydride (50 mL, 1:5 w/v) for 30 min with constant stirring. Excess acetic anhydride was evaporated to dryness under vacuum at 70 °C. The clear residue was further polymerized by melt condensation at 140 °C for 6 h under vacuum (10 mbar) with constant stirring. PHSAP was obtained as a dark-brown colored clear pasty polymer. *M*_w_ by GPC = 11 400 (dispersity = 1.96); ^1^H NMR (300 MHz, CDCl_3_) δ 7.82–7.76 (m, 1H), 7.74–7.64 (m, 1H), 7.61–7.54 (m, 2H), 5.08 (p, *J* = 6.1 Hz, 1H), 2.56 (t, *J* = 7.3 Hz, 1H), 2.42 (t, *J* = 7.4 Hz, 1H), 1.72–1.55 (m, 8H), 1.42–1.26 (m, 20H), 0.86 (t, *J* = 6.0 Hz, 5H); FTIR (cm^–1^) 3010, 2924, 2854, 1815, 1722, 1598, 1579, 1465, 1407, 1282, 1210, 1133, 1014.

#### 2.5.11 Poly(hydroxyoctanoic acid succinate) (PHOAS)

PHOAS was synthesized by reflux of HOAS with acetic anhydride followed by polymerization through melt condensation. PHOAS was synthesized by refluxing HOAS (5 g) with acetic anhydride (25 mL, 1:5 w/v) for 30 min with constant stirring. Excess acetic anhydride was evaporated to dryness under vacuum at 70 °C. The clear residue was further polymerized by melt condensation at 140 °C for 6 h under vacuum (10 mbar) with constant stirring. PHOAS was obtained as a dark-brown colored pasty polymer. *M*_w_ by GPC = 8000 (dispersity = 2.65); ^1^H NMR (300 MHz, CDCl_3_) δ 5.07 (t, *J* = 6.5 Hz, 1H), 4.50–3.78 (m, 2H), 2.88–2.57 (m, 2H), 2.05–1.72 (m, 2H), 1.55–1.03 (m, 8H), 0.88 (t, *J* = 6.0 Hz, 3H); FTIR (cm^–1^) 2955, 2927, 2860, 1827, 1747, 1458, 1378, 1360, 1170, 1062, 1033.

#### 2.5.12 Poly(hydroxydodecanoic acid succinate) (PHDDAS)

PHDDAS was synthesized by reflux of HDDAS with acetic anhydride followed by polymerization through melt condensation. PHDDAS was synthesized by refluxing HDDAS (5 g) with acetic anhydride (25 mL, 1:5 w/v) for 30 min with constant stirring. Excess acetic anhydride was evaporated to dryness under vacuum at 70 °C. The clear residue was further polymerized by melt condensation at 140 °C for 6 h under vacuum (10 mbar) with constant stirring. PHDDAS was obtained as a pale-brown colored solid. ^1^H NMR (300 MHz, CDCl_3_) δ 4.09 (t, *J* = 6.7 Hz, 2H), 2.77 (t, *J* = 6.9 Hz, 2H), 2.66 (t, *J* = 6.5 Hz, 2H), 2.45 (q, *J* = 7.3 Hz, 2H), 1.75–1.53 (m, 4H), 1.45–1.24 (m, 14H); FTIR (cm^–1^) 2916, 2849, 1816, 1744, 1464, 1417, 1320, 1184, 1125, 1045.

### 2.6 Storage Stability Studies

Polyanhydrides were investigated for their storage stability at room temperature. All of the samples (∼50 mg, in duplicate) were kept at room temperature (∼25 °C) under a nitrogen atmosphere. The change in the molecular weight was regularly recorded for 3 months using GPC, and the results were compared with PSA and P(SA-RA).

### 2.7 Hydrolytic Degradation Studies

Twelve polymer samples (∼100 mg, in duplicate), PSA, PDDA, P(SA-RA), P(SA-HAS), PRAS, PRAM, PRAP, PHSAS, PHSAM, PHSAP, PHOAS, and PHDDAS, were analyzed for hydrolytic degradation studies. Each sample was taken in a 1 mL Eppendorf tube containing 1 mL of a 0.1 M phosphate buffer solution (PBS, pH 7.4). Then, all of the samples were kept at 37 °C with constant shaking (100 rpm). In total, five independent sample sets were used to study hydrolysis at different intervals such as 1, 3, 7, 14, and 30 days. The buffer was replaced at regular intervals. At each time point (after 1, 3, 7, 14, and 30 days), the buffer was removed from polymer samples and lyophilized. The hydrolysis was monitored and compared with the initial polymers by FTIR spectroscopy and molecular weight by GPC.

### 2.8 *In Vitro* Drug Release Studies

Nine injectable pasty polymers, P(SA-RA), P(SA-HAS), PRAS, PRAM, PRAP, PHSAS, PHSAM, PHSAP, and PHOAS, were investigated for their *in vitro* drug release properties using ibuprofen as a model drug. A homogeneous injectable formulation was obtained by triturating the pasty polymers with ibuprofen (10% (w/w)) powder. Each formulation (∼200 mg) was placed in the bottom of a 15 mL Eppendorf tube containing 10 mL of a 0.1 M phosphate buffer solution (PBS, pH 7.4). Then, all of the samples were kept at 37 °C with constant shaking (100 rpm). The solutions were taken out after 5 h, 1, 3, 7, 14, 21, 28, and 35 days without disturbing the formulation. After removing the medium, a fresh buffer solution was added at all time points. The samples were analyzed for ibuprofen quantity using UV by measuring the absorption at 264 nm. The collected samples were diluted as needed during UV analysis. The percentage quantity of ibuprofen released at each time point was determined using a calibration curve. All of the experiments were conducted in triplicate.

## 3 Results and Discussion

### 3.1 Design of Diverse Alternative Polyanhydrides

**Figure 1:**
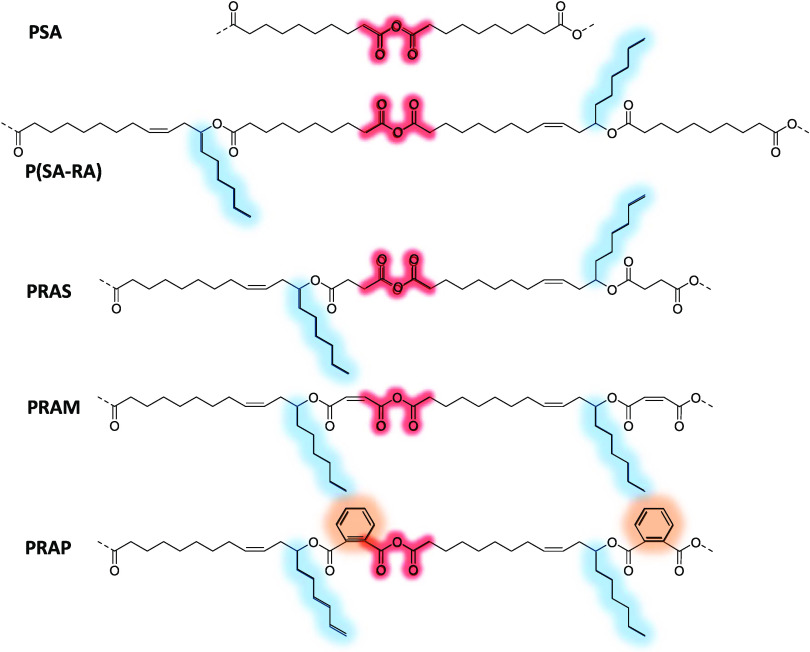
Design of poly(ester-anhydride)s such as PRAS, PRAM, and PRAP from RA and succinic, maleic, and phthalic anhydrides, respectively. The alternating architecture was designed by the polymerization of ester-diacids prepared from RA with anhydrides. The hydrophobic side chains and phenyl moieties are designed nearby to anhydride bonds to improve the hinderance to hydrolytic cleavage and anhydride interchange.

We recently reported the alternative P(SA-RA), poly(ester-anhydride), based on the SA and RA (weight ratio 30:70) with improved stability and shelf-life that is stable at 25 °C for more than 18 months.^19^ The alternating architecture and hydrophobic side chains of P(SA-RA) hinder hydrolytic cleavage and anhydride interchange. In this report, we designed alternating architecture by the polymerization of ester-diacids prepared from RA with anhydrides. The hydrophobic side chains are designed closer to anhydride bonds to improve the hinderance to hydrolytic cleavage and anhydride interchange as shown in Figure 1. We aimed PRAS, a poly(ester-anhydride) with the hydrophobic side chains, very close to the anhydride bond compared to P(SA-RA). Phenyl moiety (PRAM) is also designed near the anhydride bonds in addition to the hydrophobic side chain.

**Figure 2:**
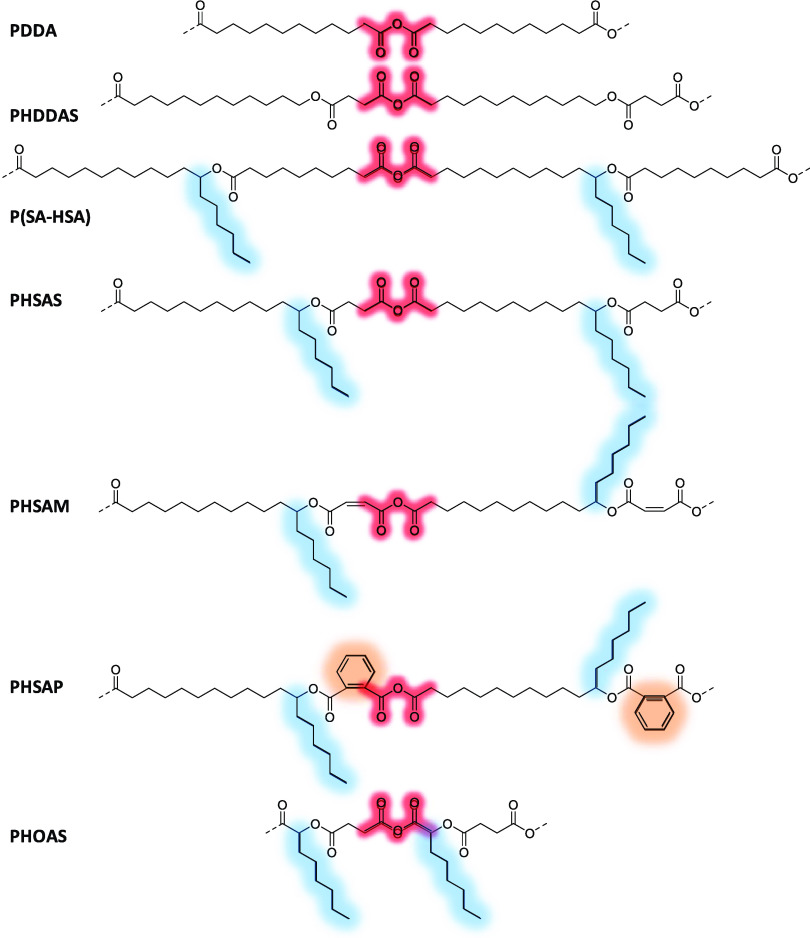
Design of poly(ester-anhydride)s such as PHSAS, PHSAM, and PHSAP from HSA and succinic, maleic, and phthalic anhydrides, respectively, and PHOAS from HOA and succinic anhydride. These polyanhydrides were designed to investigate the effect of ester bonds (PHDDAS), phenyl moieties (PHSAP), hydrophobic side chains, and their distance from anhydride bonds (PHOAS).

The various polyanhydrides were designed to investigate the effect of ester bonds, hydrophobic side chains, phenyl moieties, and their distance from anhydride bonds on their stability and properties (Figure 2). PDDDA and P(SA-HSA) (30:70) were used instead of PSA and P(SA-HSA) (30:70) to keep the same length in the polymeric backbone chain. PHDDAS was designed to evaluate the effect of ester bonds in poly(ester-anhydride) compared to only polyanhydride (PDDDA). PHSAS was designed to investigate the effect of decreasing the polymeric backbone chain length, thereby making hydrophobic side chains closer to anhydride bonds. In PHSAP, phenyl moieties were incorporated in addition to hydrophobic side chains to study their properties. Finally, PHOAS was designed to reduce the polymeric backbone chain length and to make hydrophobic side chains very close to anhydride bonds.

### 3.2 Synthesis of Designed Polyanhydrides

**Scheme 1:**
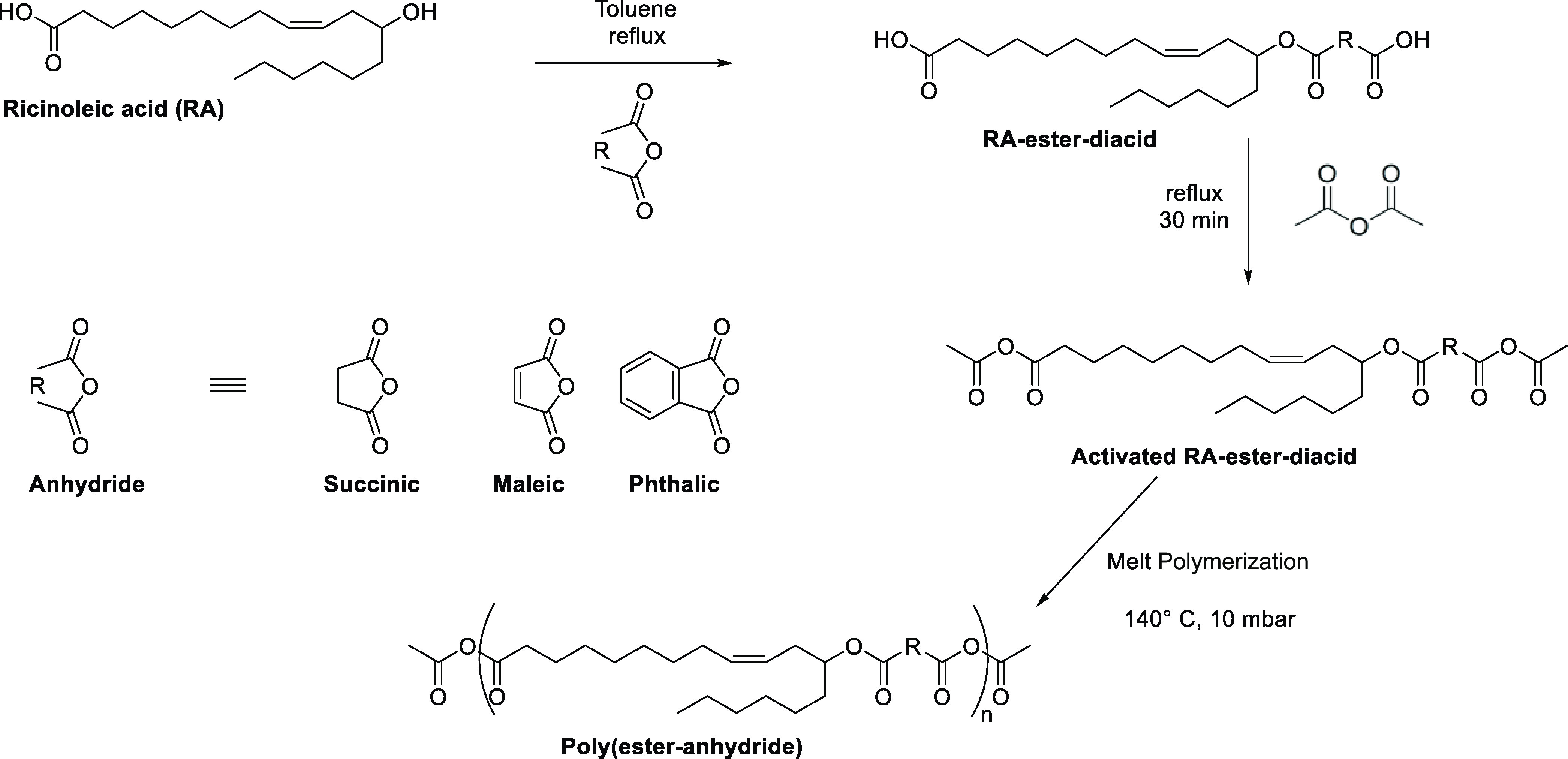
Synthetic Methodology to Convert Hydroxy Acid to Poly(ester-anhydride) Using Anhydride First step: Conversion of RA to ester-diacid by the esterification reaction with succinic, maleic, and phthalic anhydrides using toluene as a solvent at reflux. Second step: RA-ester-diacid is activated using acetic anhydride. Third step: poly(ester-anhydride)s are obtained by melt condensation.

The detailed synthetic methodology is given in Scheme 1. In the first step, various hydroxy acids are converted to ester-diacids by the esterification reaction with different anhydrides using toluene as a solvent at 90 °C. Then, the ester-diacids are activated using acetic anhydride. Finally, poly(ester-anhydride)s are obtained by melt condensation. Synthesis of ester-diacid was optimized using RA and succinic, maleic, and phthalic anhydrides.

### 3.3 Synthesis of Ester-Diacid Monomers

**Scheme 2:**
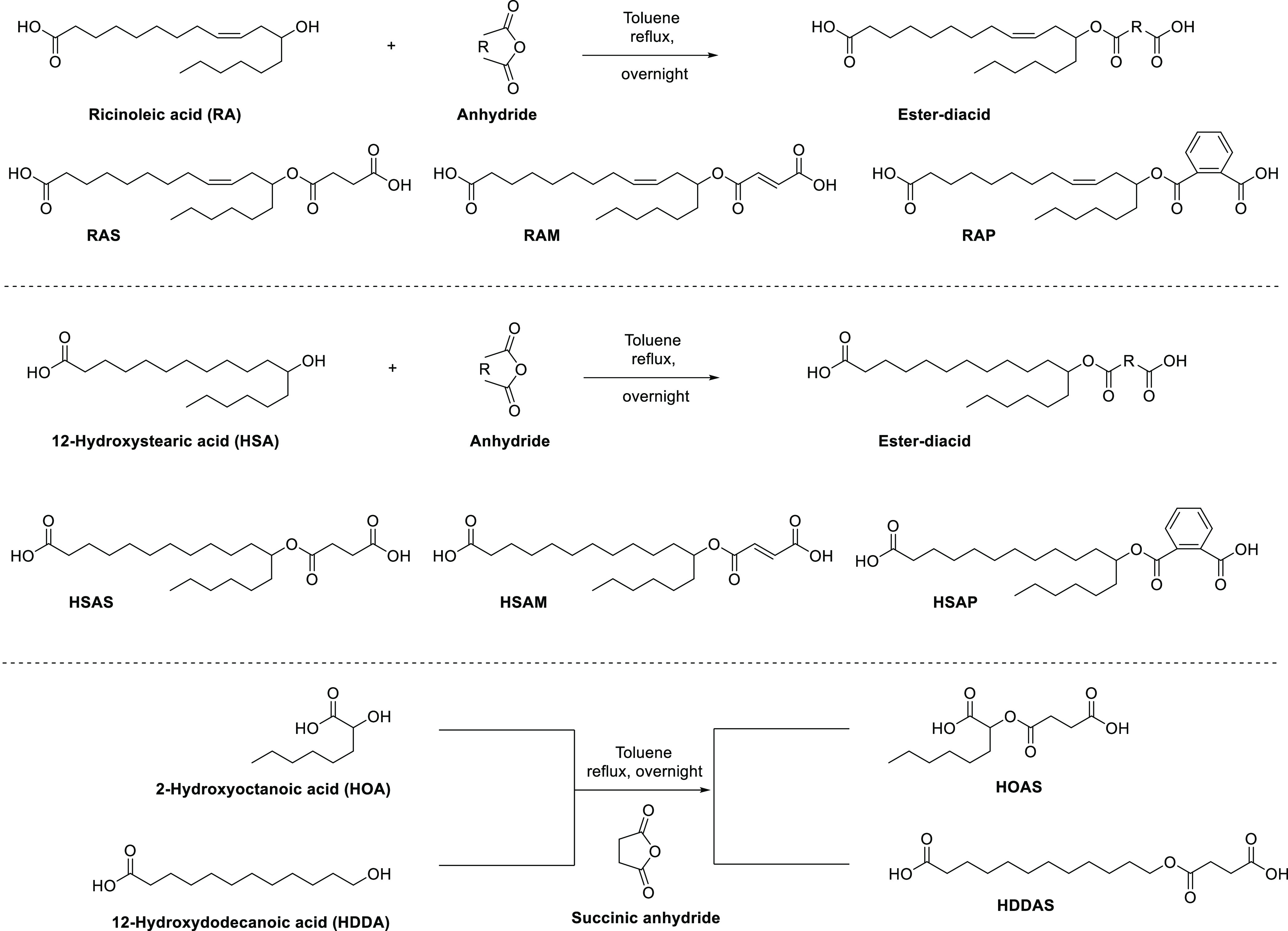
Synthetic Methodology to Convert Hydroxy Acid into Ester-Diacid Using Anhydride Conversion of various hydroxy acids into ester-diacids by esterification reaction with succinic, maleic, and phthalic anhydrides using toluene as a solvent at reflux.

RA was reacted with an excess quantity of anhydrides at 90 °C in toluene for the complete conversion of RA to avoid purification (Scheme 2). If an excess amount of anhydride is taken, it must be removed by washing with water. However, only succinic anhydride is highly reactive with water. Maleic and phthalic anhydrides, however, are less reactive with water. Thus, anhydrides were removed by heating with water at 50 °C for 30 min after the complete consumption of RA. Before the addition of water, toluene was removed to avoid the formation of an emulsion. The reaction progress was monitored by TLC using vanillin stain. Ester-diacids such as RAS, RAM, and RAP were obtained as a viscous liquid. Subsequently, this protocol extended to other hydroxy acids. HSA was reacted with succinic, maleic, and phthalic anhydride to obtain ester-diacids such as HSAS, HSAM, and HSAP, respectively, as solids. HOA and HDDA were reacted with succinic anhydride to obtain ester-diacids such as HOAS (liquid) and HDDAS (solid), respectively.

### 3.4 Synthesis of poly(ester-anhydride)s

**Scheme 3:**
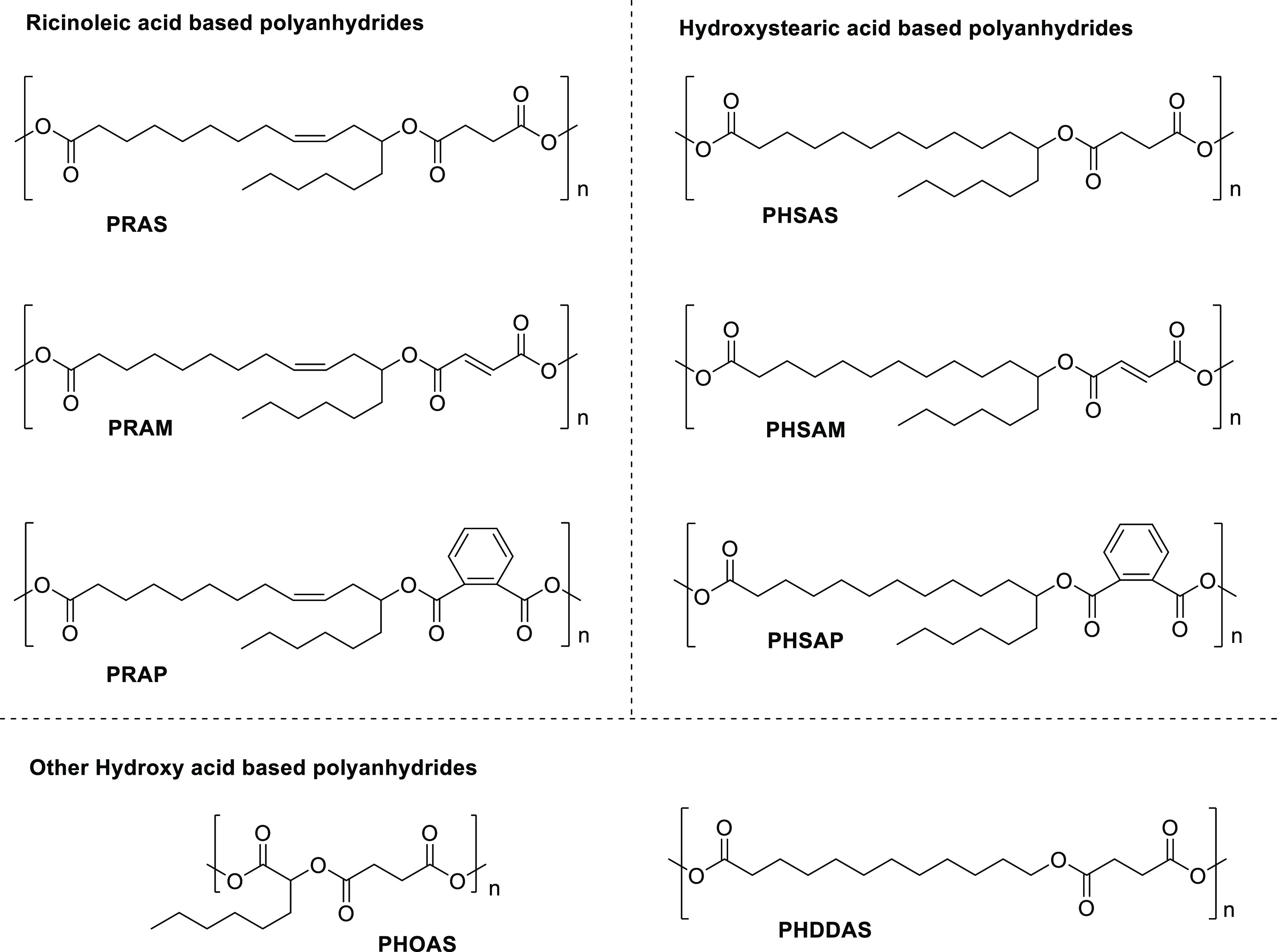
Various Poly(ester-anhydride)s Synthesized from Ester-Diacids by Activation Using Acetic Anhydride Followed by Melt Condensation

After the synthesis of all of the monomers such as RAS, RAM, RAP, HSAS, HSAM, HSAP, HOAS, and HDDAS, the synthesis of poly(ester-anhydride) such as PRAS, PRAM, PRAP, PHSAS, PHSAM, PHSAP, PHOAS, and PHDDAS was performed by melt condensation (Scheme 3). First, the ester-diacid monomers were activated through the reflux with 1:5 w/v acetic anhydride for 30 min. Excess acetic anhydride was evaporated to dryness under vacuum at 70 °C. The clear residue was further polymerized by melt condensation at 140 °C for 6 h under vacuum (10 mbar) with constant stirring, which provides poly(ester-anhydride)s as the injectable pasty polymer. The injectability of the pasty polymers was determined by placing 1 mL of the polymer in a 1 mL BD Luer-Lok syringe with an inner diameter of 5 mm with a 21G needle and pressed at a rate of 20 mm/min. All pasty polymers passed this test.

PSA, PDDDA, P(SA-RA), and P(SA-HSA) were prepared to compare the stability and properties of the newly designed and synthesized poly(ester-anhydride)s. PSA and PDDDA were prepared using SA and DDDA, respectively, through melt condensation at 140 °C for 6 h under vacuum (10 mbar). Also, P(SA-RA) and P(SA-HSA) were prepared by the reaction of PSA with RA and HSA, respectively, using 30:70 weight ratio. The synthesis involved an esterification reaction of RA or HAS onto PSA to form carboxylic acid-terminated oligomers followed by anhydride polymerization.

### 3.5 Characterization

#### 3.5.1 FTIR

**Figure 3:**
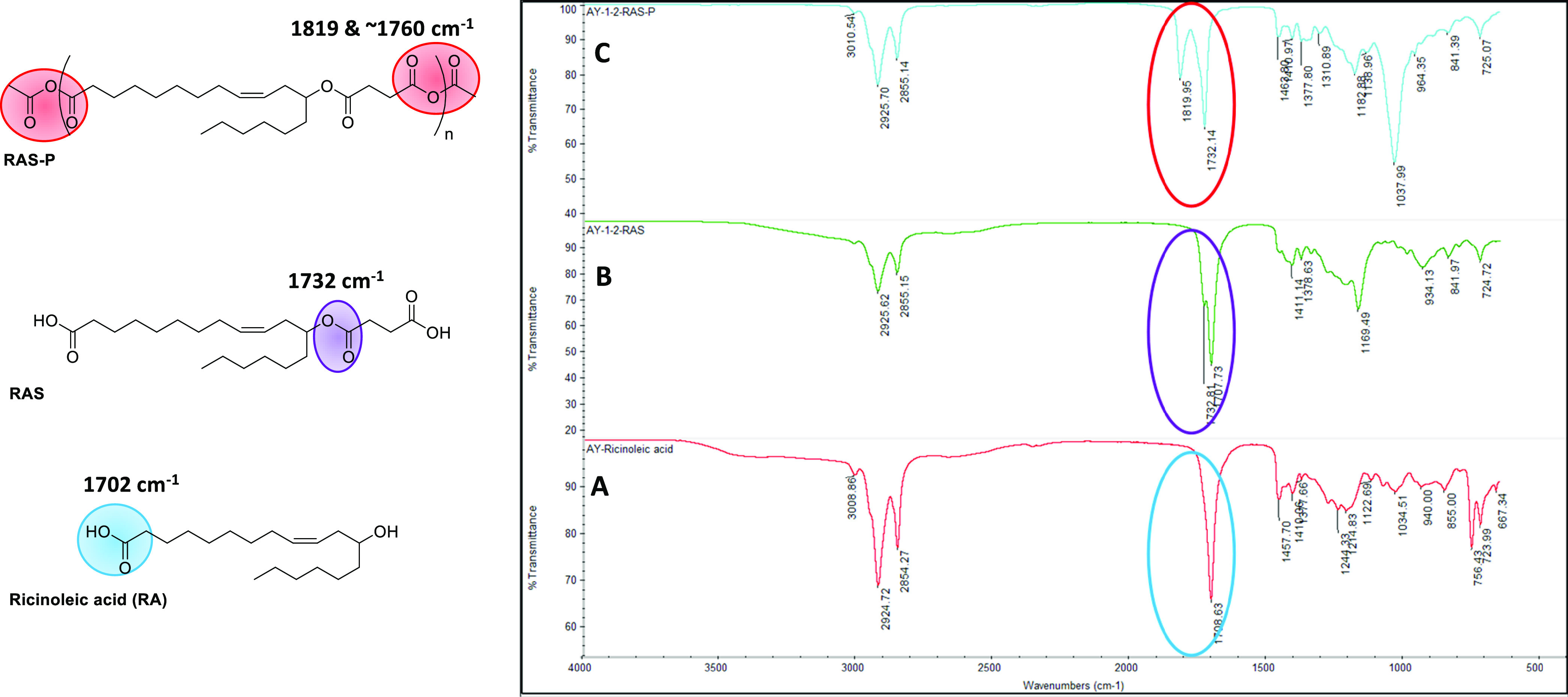
Comparison of FTIR spectrum reactant, monomer, and polymer: (A) RA; (B) formation of RAS from RA and succinic anhydride; and (C) final formation of polyanhydride (PRAS).

The FTIR spectra of RA, monomer, and polymer are presented in Figure 3. The characteristic stretching frequency at 1702 cm^–1^ corresponds to the C=O (acid) of RA. After the reaction of RA with succinic anhydride, the formation of RAS was confirmed by the appearance of a sharp C=O (ester) band at 1732 cm^–1^. Then, the ester-diacid was polymerized and poly(ester-anhydride) was confirmed by the characteristic bands at 1819 and 1760 cm^–1^ for C=O (anhydride) of PRAS.

#### 3.5.2 ^1^H NMR

**Figure 4:**
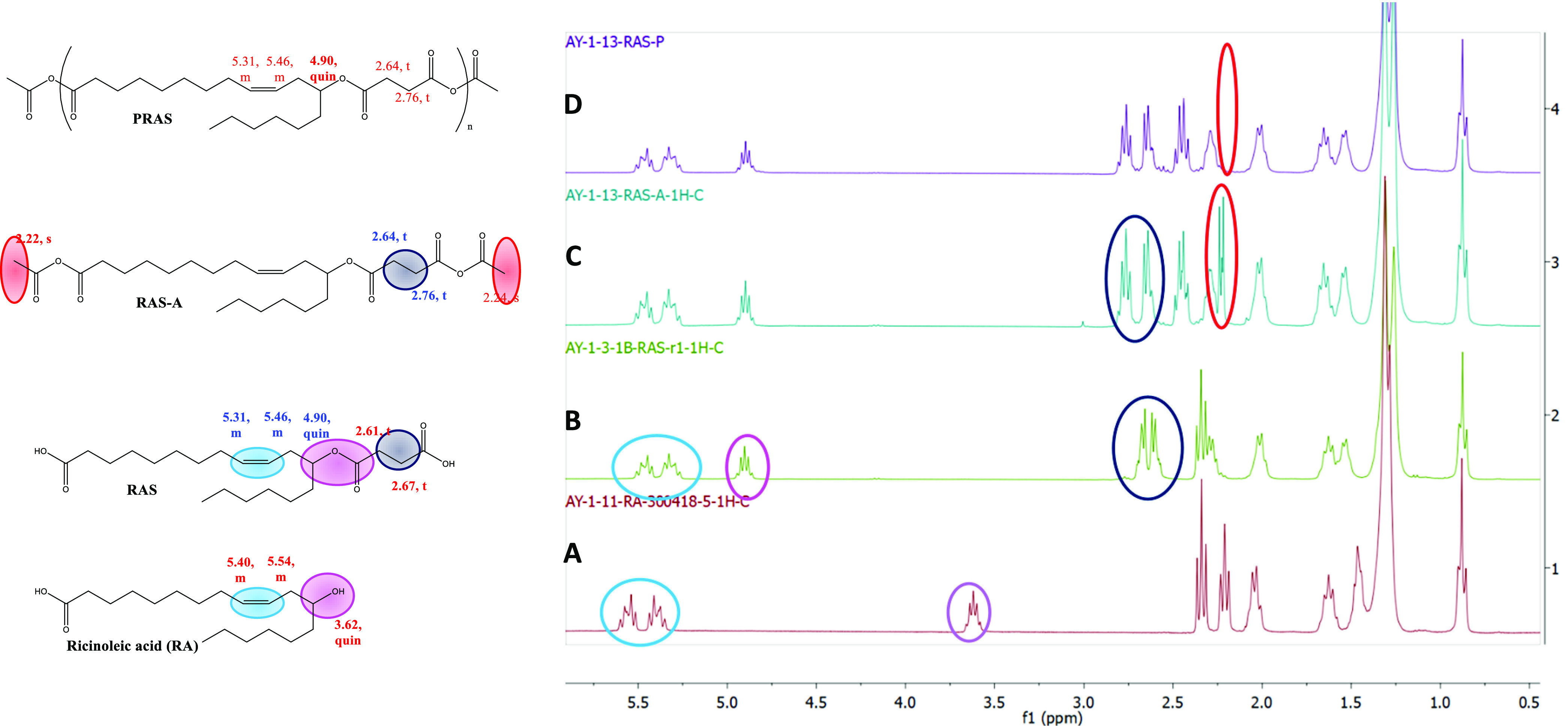
Comparison of ^1^H NMR spectrum in CDCl_3_ to confirm the completion of the reaction and the structure of the monomer and polymer: (A) RA; (B) formation of RAS from RA and succinic anhydride; (C) activation of RAS diacid using acetic anhydride; and (D) final formation of polyanhydride (PRAS).

The progress of the monomer and polymer synthesis was monitored by NMR. In addition, the structure of synthesized monomers and polymers was confirmed by NMR spectroscopy (Figure 4). In ^1^H NMR of RA, the characteristic pentet peak at 3.62 ppm is observed for CH–OH. Also, the double bond protons are observed at 5.54 and 5.40 ppm. When RA reacted with succinic anhydride, the characteristic pentet peak of RA at 3.62 ppm is shifted to 4.90 ppm in RAS. In addition, two new peaks for succinate CH_2_ protons are detected at 2.67 and 2.61 ppm. During the activation of RAS diacid with acetic anhydride, the peaks at 2.34 and 2.22 ppm for CH_3_ show the confirmation of acetylation. The absence of acetylated CH_3_ peaks at 2.34 and 2.22 ppm in the final polymer PRAS confirms the completion of polymerization.

### 3.6 Molecular Weight by GPC

The molecular weight of polyanhydrides was determined using GPC (Table 1). The polyanhydrides were obtained in the molecular weight ranges from 8000 to 23 600 Da. Lesser molecular weight was observed for PRAP and PHSAP due to the steric hindrance of the phenyl moiety near the active site acid. PHOAS exhibits the least molecular weight among all of the polyanhydrides due to the steric hindrance of the long side chain present in the vicinity to both active site acids.

**Table 1:** Molecular Weight of Synthesized Polymers Analyzed by GPC^a^

no.	name	number avg. molecular weight (*M*_n_) Da	weight avg. molecular weight (*M*_w_) Da	dispersity
**1**	poly(sebacic acid) (PSA)	4800	10 600	2.2
**2**	poly(dodecanedioic acid) (PDDDA)	5200	12 000	2.3
**3**	poly(sebacic acid-ricinoleic acid) (P(SA-RA))	5000	11 500	2.3
**4**	poly(sebacic acid-hydroxystearic acid) (P(SA-HSA))	6200	13 100	2.1
**5**	poly(ricinoleic acid succinate) (PRAS)	8000	14 700	1.83
**6**	poly(ricinoleic acid maleate) (PRAM)	6400	11 900	1.87
**7**	poly(ricinoleic acid phthalate) (PRAP)	4500	8400	1.86
**8**	poly(hydroxystearic acid succinate) (PHSAS)	7800	19 100	2.44
**9**	poly(hydroxystearic acid maleate) (PHSAM)	8800	23 600	2.69
**10**	poly(hydroxystearic acid phthalate) (PHSAP)	5800	11 400	1.96
**11**	poly(hydroxyoctanoic acid succinate) (PHOAS)	3000	8000	2.65

a Samples (∼2 mg) were dissolved in 2 mL of CHCl_3_ (HPLC grade). GPC was performed using a column with a molecular-weight range of 50–100K Da. The molecular weights were determined relative to polystyrene standards.

### 3.7 Storage Stability Studies

Generally, poly(ester-anhydride)s are unstable at room temperature. A sharp decline in molecular weight has been observed at room temperature in the previous reports. The molecular weight of polyanhydrides was stable for only 1 month and declined to about one third after 6 months at 4 °C. In addition, they were stable merely for a few days at room temperature. This instability at room temperature raises a practical problem with storage and handling.^27^ Moreover, reportedly, the block and random (SA-RA) copolymer was unstable at room temperature.^28^ There were blocks of SA units along the chain, which makes it vulnerable to rapid anhydride interchange. Thus, when polyanhydrides were stored at room temperature, a sharp decline in *M*_w_ was noticed. However, the recently reported alternating P(SA-RA) (weight ratio 30:70) copolymer exhibits stable molecular weight for 18 months.^19^ RA side chains of alternate RA-SA polymer obstruct anhydride interchange and hydrolytic degradation by steric hindrance.

**Figure 5:**
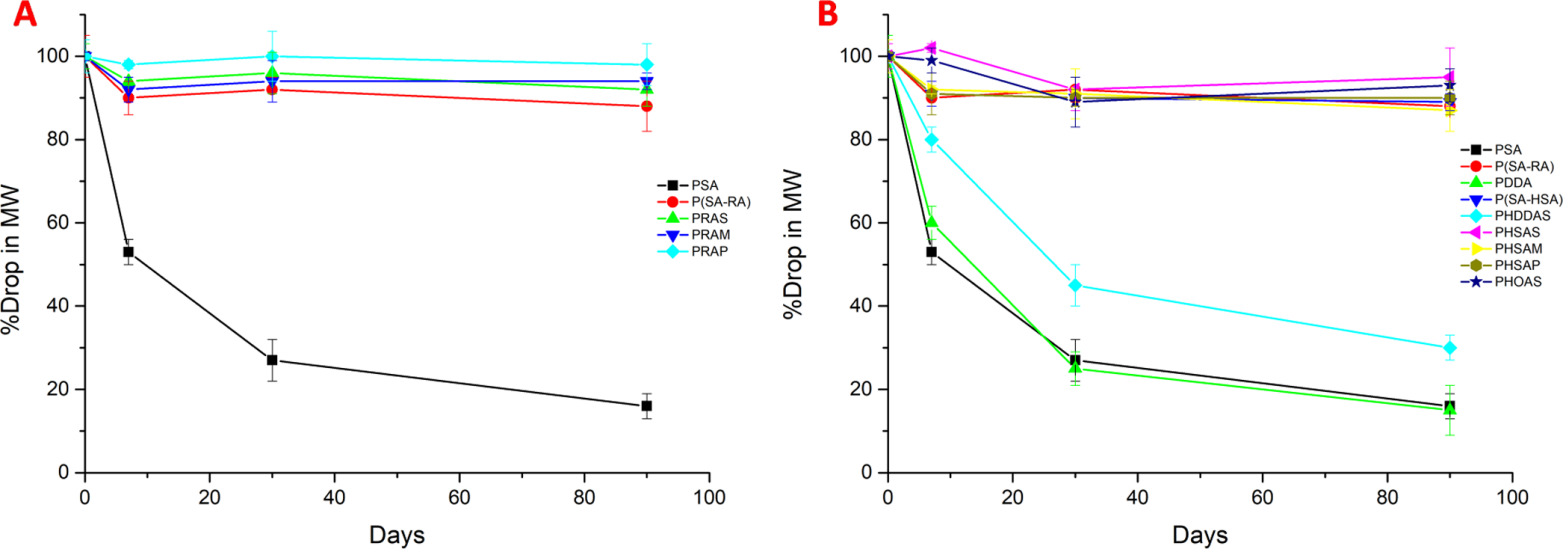
Storage stability studies of polyanhydrides at room temperature for 3 months. (A) Relative stability of ricinoleic acid-based polyanhydrides with PSA and P(SA-RA). (B) Relative stability of polyanhydrides with PSA and P(SA-RA) from hydroxy acid with different chain lengths. The molecular weights are an average of at least two independent molecular-weight determinations of two samples.

In this study, polyanhydride samples were packed under dry nitrogen in sealed tubes. Then, the polymer samples were stored at room temperature (∼25 °C) for 3 months. At each time-point (7 days, 1 month, and 3 months), GPC analysis was conducted to determine the change in the molecular weight. The results were compared with PSA and alternating P(SA-RA) (weight ratio 30:70). The molecular weight of the tested poly(ester-anhydride)s with a shorter chain length compared to P(SA-RA) was constant for 3 months (Figure 5). The side chain present in the closer vicinity to the anhydride bonds offers improved stabilization, hindering hydrolytic cleavage and anhydride interchange.^29^ This essential storage stability permits ease of handling and formulation of poly(ester-anhydride)s for drug delivery under common conditions. Figure 5 shows the stability comparison of poly(ester-anhydride)s with PSA and P(SA-RA). It should be noted that shelf-life stability is related to change in the molecular weight under dry conditions where the polymers may be affected by traces of humidity entrapped during polymer synthesis. There is a lipid chain that masks the anhydride bond from hydrolysis in an alternating structure of the polymers near any anhydride bond along the polymer chain.

### 3.8 Hydrolytic Degradation Studies

**Figure 6:**
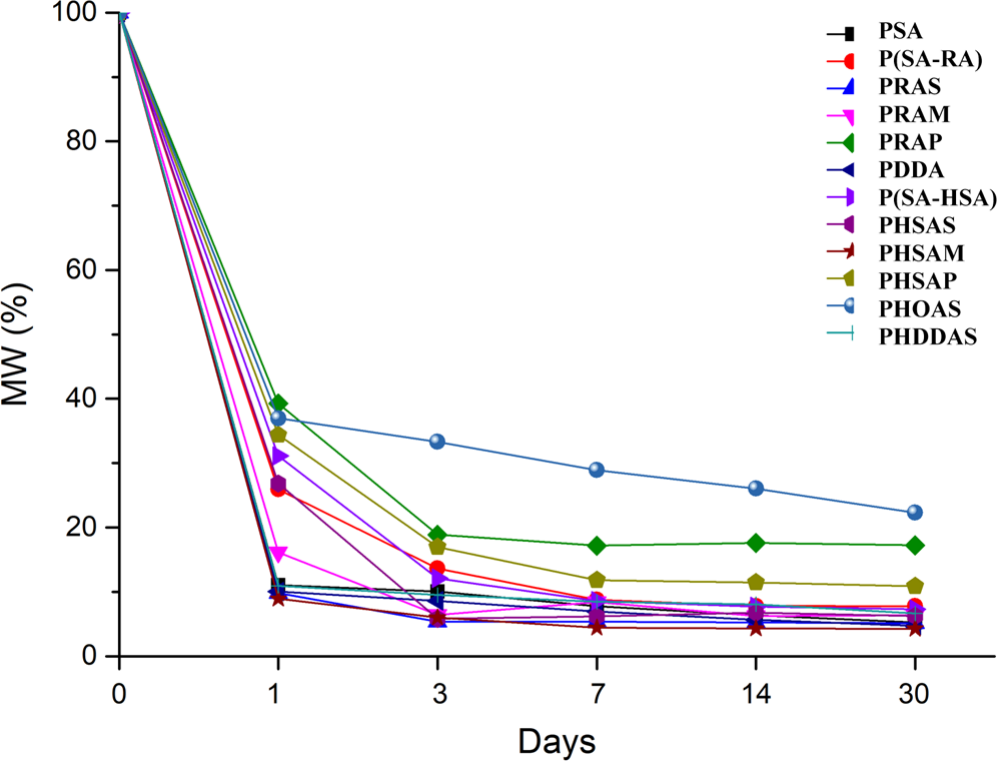
Hydrolytic stability studies of polyanhydrides in phosphate buffer pH 7.4 at 37 °C. Polymer samples were taken at the regular time point and molecular weight was determined by GPC. The data represent an average of at least two independent molecular-weight determinations and samples with a standard deviation less than 10% of each data point.

The synthesized injectable pasty polyanhydrides were analyzed for hydrolytic degradation studies and compared the results with PSA and P(SA-RA). The molecular-weight change of the polyanhydrides was measured at each time point (after 1, 3, 7, 14, and 30 days) by performing GPC analysis. The results were provided in Figure 6. The rate of hydrolysis of novel poly(ester-anhydride)s is slower when compared with PSA. As we reported previously, poly(ester-anhydride)s undergo hydrolytic degradation in two stages.^19,30^ At first, the anhydride bonds of poly(ester-anhydride) are cleaved, quickly releasing the diacid units, followed by the slow degradation of oligoesters.^18,31^ After the first day, seven polyanhydrides such as P(SA-RA), P(SA-HAS), PRAM, PRAP, PHSAS, PHSAP, and PHOAS exhibit higher *M*_w_ than PSA. After 3 days, five polyanhydrides such as P(SA-RA), P(SA-HAS), PRAP, PHSAP, and PHOAS show higher *M*_w_ than PSA. Interestingly, after 7, 14, and 30 days of GPC analysis, three polyanhydrides, PRAP, PHSAP, and PHOAS, still display better *M*_w_ than PSA and P(SA-RA). These results reveal that the phenyl moiety present in PRAP and PHSAP reduces the hydrolysis of anhydride bonds. PHOAS demonstrates the highest *M*_w_ of all of the tested polymers and exhibits a moderate change from 1 to 30 days with *M*_w_ staying around 30%. This clearly shows that the presence of a side chain closer to the anhydride bond significantly decreases the hydrolysis. The hydrolytic degradation is different from exposing the polymer to endless amounts of water that attack the anhydride bonds, and thus the differences in degradation are less significant.

### 3.9 *InVitro* Drug Release Studies

**Figure 7:**
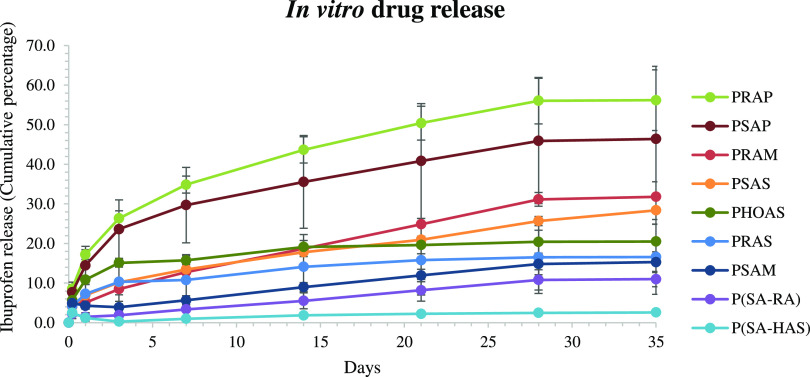
*In vitro* release of ibuprofen in 0.1 M phosphate buffer (pH 7.4) at 37 °C mixed at 100 rpm. The amount of ibuprofen was calculated using UV. The percentage errors are calculated from an average of three observations.

The synthesized injectable pasty polymers such as P(SA-RA), P(SA-HAS), PRAS, PRAM, PRAP, PHSAS, PHSAM, PHSAP, and PHOAS were examined for their *in vitro* drug release pattern using ibuprofen as a model drug (Figure 7). The results reveal that the nature of the polymer influences the ibuprofen release from the polymer matrix. Polymers with aromatic units such as PRAP and PHSAP exhibit sustained release of ibuprofen following the ∼8% primary burst release of the drug.^19^ As a result of the hydrolytic cleavage of the anhydride bonds, poly(ester-anhydride) has this initial characteristic burst release pattern. PRAP and PHSAP polymers released more than 50 and 40% of ibuprofen over a period of 28 days, respectively. The polymer with better stability than hydrolytic degradation of PHOAS showed marginally increased release compared to P(SA-RA). This study demonstrates that the nature of the oligomers formed after hydrolytic degradation affects the release of ibuprofen from the tested polymers. These short oligomers were formed due to the hydrolytic degradation of poly(ester-anhydride), controlling the release of the drug in a sustained manner.^27^

## 4 Conclusions

The alternating architecture and hydrophobic side chains of P(SA-RA) hinder hydrolytic cleavage and anhydride interchange. We designed an alternating architecture by the polymerization of ester-diacids prepared from ricinoleic or other hydroxy acids with anhydrides such as succinic, maleic, and phthalic anhydrides. In addition, the hydrophobic side chains are designed closer to anhydride bonds to improve the hinderance to hydrolytic cleavage and anhydride interchange. The series of poly(ester-anhydride)s such as PRAS, PRAM, PRAP, PHSAS, PHSAM, PHSAP, PHOAS, and PHDDAS were synthesized to investigate the effect of ester bonds, hydrophobic side chains, phenyl moieties, and their distance from anhydride bonds on their stability and properties. In the first step, hydroxy acid is converted to ester-diacid by the esterification reaction with anhydrides. Then, the ester-diacid is activated using acetic anhydride. Finally, the injectable pasty poly(ester-anhydride)s are obtained by melt condensation. PSA, PDDDA, P(SA-RA), and P(SA-HSA) were used to compare the stability and properties. The reaction progress and structure of the monomer and polymer were monitored by NMR and FTIR. The molecular weight of the polyanhydrides was determined using GPC. The polyanhydrides were obtained in an excellent molecular-weight range. Polyanhydrides were investigated for their storage stability at room temperature (∼25 °C) under a nitrogen atmosphere for 3 months using GPC and compared the results with PSA and P(SA-RA). The molecular weight of the tested poly(ester-anhydride)s with a shorter chain length compared to P(SA-RA) was stable for 3 months. Polyanhydrides were analyzed for hydrolytic degradation studies by performing GPC analysis. These results reveal that the phenyl moiety present in PRAP and PHSAP reduces the hydrolysis of anhydride bonds. Notably, PHOAS demonstrates the higher *M*_w_ of all of the tested polymers. The results show that the presence of hydrophobic side chains, phenyl moieties, and their distance from anhydride bonds significantly decreases hydrolysis. The synthesized injectable pasty polymers were analyzed for their *in vitro* drug release pattern using ibuprofen. Polymers with aromatic units such as PRAP and PHSAP demonstrate sustained release that displayed more than 50 and 40% of ibuprofen over a period of 28 days, respectively. These polymers have potential use as injectable biodegradable polymers for tissue augmentation and drug release carriers.
